# Cyanotoxin Analysis of Air Samples from the Great Salt Lake

**DOI:** 10.3390/toxins15110659

**Published:** 2023-11-15

**Authors:** James S. Metcalf, Sandra Anne Banack, Paul Alan Cox

**Affiliations:** 1 Brain Chemistry Labs, Jackson, WY 83001, USA; sandra@ethnomedicine.org (S.A.B.); paul@ethnomedicine.org (P.A.C.); 2 Department of Biological Sciences, Bowling Green State University, Bowling Green, OH 43403, USA

**Keywords:** cyanobacteria, saline, sediment, climate change, exposure, dust

## Abstract

The Great Salt Lake in Utah is the largest saline lake in the Western hemisphere and one of the largest terminal lakes in the world. Situated at the eastern edge of the Great Basin, it is a remnant of the freshwater Lake Bonneville whose water level precipitously lowered about 12,000 years ago due to a natural break in Red Rock pass to the north. It contains a diverse assemblage of cyanobacteria which vary spatially dependent on salinity. In 1984, the waters of the Great Salt Lake occupied 8500 km^2^. Nearly four decades later, the waters occupy 2500 km^2^—a reduction in surface area of 71%. With predominantly westerly winds, there is a potential for the adjacent metropolitan residents to the east to be exposed to airborne cyanobacteria- and cyanotoxin-containing dust. During the summer and fall months of 2022, air and dried sediment samples were collected and assessed for the presence of BMAA which has been identified as a risk factor for ALS. Collection of air samples equivalent to a person breathing for 1 h resulted in BMAA and isomers being found in some air samples, along with their presence in exposed lakebed samples. There was no clear relationship between the presence of these toxins in airborne and adjacent lakebed samples, suggesting that airborne toxins may originate from diffuse rather than point sources. These findings confirm that continued low water levels in the Great Salt Lake may constitute an increasing health hazard for the 2.5 million inhabitants of communities along the Wasatch Front.

## 1. Introduction

As the largest saline lake in the Western hemisphere, the Great Salt Lake (GSL) has played an important role in the ecosystems, economy, and history of the Great Basin. Food webs in this saline lacrustine ecosystem are founded on planktonic organisms, particularly salt-tolerant green algae such as *Dunaliella salina* and various species of cyanobacteria, which are largely distributed according to salinity [1,2,3,4]. *Aphanothece halophytica* proliferates in the highly saline northern arm (27% saline), while *Nodularia spumigena* occurs in the less saline south (6–10%; [5,6]). Species of *Oscillatoria* and *Phormidium* also occur [7,8]. It has been experimentally shown that *Aphanothece halophytica* is suppressed in the presence of *Nodularia spumigena*. Conversely, *N. spumigena* cannot survive the hypersaline conditions in the Northern arm where *Aphanothece halophytica* dominates; thus, both abiotic and biotic factors determine the distribution of cyanobacteria in the lake [9]. Historically, other cyanobacterial species have been recorded in the lake including *Coccochloris elebans* and *Euhalothece* spp. [10,11]. Built upon the halophilic plankton foundation of the lacrustine ecosystem are salt tolerant animals, including *Prorodon utahensis*, a protist which flourishes in the hypersaline waters, whose cilia provide its tiny body—scarcely the width of a human hair—with sufficient locomotion to feed on cyanobacteria and *Dunaliella* [12,13,14]. The brine shrimp *Artemia salina* and the brine flies of the genus *Ephydra* also feed on cyanobacteria [15,16]. They in turn provide food for vast populations of migratory birds including green-eared grebes (which are able to use 87–97% of the gross energy of these small animals), northern shovelers, red-necked phalaropes, and green-wing teal [17,18,19,20]. Due to the persistent drought and increased diversion of water from the Bear River, Jordan River, and other tributaries, lake levels have reached historic lows, such that this ecosystem is in danger of collapse [21].

Although cyanobacteria form the basis of food chains in the Great Salt Lake, they are better known for their ability to produce a range of toxic substances (cyanotoxins). Included in these are hepatotoxins (microcystins, nodularins), cytotoxins (cylindrospermopsins) and neurotoxins (saxitoxins, anatoxin-a, guanitoxin, BMAA and isomers) with both short-term and long-term adverse health effects [22,23,24,25,26,27,28,29,30,31,32,33,34,35,36,37,38,39,40]. In addition to affecting salinity, the low water levels in the lake result in the exposure of cyanobacterial stromatolites, microbiolites and dried lake beds. Coupled with the potential for dust production from a predominantly westerly wind, the possibility exists for crusts and other dried lakebed material to become airborne. This has the potential to adversely affect human populations, both short- and long-term [41].

Increasingly, research into the occurrence of cyanobacteria in terrestrial environments is revealing the importance of these organisms in the development of desert habitats. Cyanobacteria are often pioneering organisms in desert environments and can cover a significant area, such as in Qatar where cyanobacterial crusts cover up to 85% of the land surface, and when disturbed can produce airborne cyanobacteria which can negatively impact human health [42,43,44,45]. Toxin analysis of such desert crusts has shown the presence of BMAA, guanitoxin (anatoxin-a(S)) and microcystins [44] with exposure to BMAA in desert dust considered as a possible cause of the spike in ALS cases among military personnel deployed during the first Persian Gulf War [42]. In addition, due to the constituents of the exposed lakebed, there are additional toxic components, such as heavy metals, which may exacerbate the toxicity of any airborne samples from the Great Salt Lake. Since both mercury and BMAA bioaccumulate in aquatic ecosystems, there may be increased peril to components of the adjacent terrestrial ecosystems including hunters of wildfowl [46,47,48]. This combination of methylmercury with BMAA is of further concern in that these two toxins are synergistic in their neurotoxicity, increasing the potential risk to human health [49].

The purpose of our study was to take air and ground samples from exposed lake beds around the eastern and southern parts of the GSL and assess the presence of a number of known cyanotoxins to estimate the potential risk to human health.

## 2. Results

**Figure 1:**
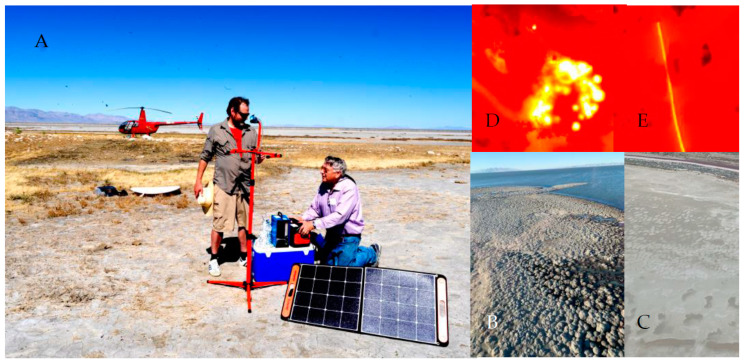
Air sampling device being set up by Metcalf and Cox on dried lakebed (**A**). Example aerial photographs of the mats (**B**,**C**) and fluorescence microscopy of coccoid (**D**) and filamentous (**E**) cyanobacteria.

**Figure 2:**
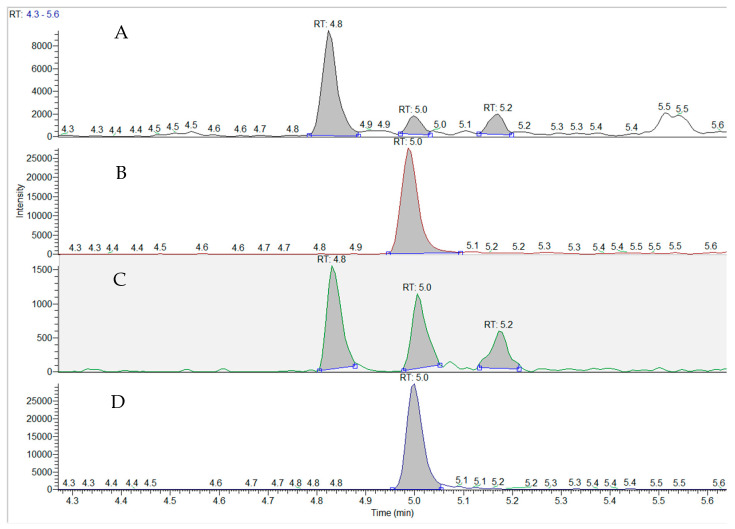
Triple quadrupole mass spectrometric analysis of BMAA in an impinger air sample from the Great Salt Lake in comparison with a BMAA standard. The air sample was taken at site 7 (see Figure 1) in October 2023. (**A**), Air sample TIC at *m*/*z* 459.2 (double derivatized AQC-BMAA, AEG and DAB); (**B**), Air sample TIC at *m*/*z* 464.25 (AQC-derivatized internal BMAA standard); (**C**), standard TIC at *m*/*z* 459.2 (double derivatized AQC-BMAA, AEG and DAB); (**D**), standard TIC at *m*/*z* 464.25 (AQC-derivatized internal BMAA standard).

Using a helicopter allowed a large part of the eastern and southern areas of the Great Salt Lake to be sampled and provided time to take adequate air samples at two sites in August and three sites in September and October 2022 (Figure 1). Fluorescence microscopic assessment of dried mats showed the presence of filamentous and coccoid cyanobacteria, although identification of the cyanobacteria was difficult due to the fact that these mats were dried. The air sampling was carried out such that the amount of air that passed through the filter to retain particulates and the impinger for free compounds was equivalent to an average person breathing for 1 h (Table 1). August was the only month when BMAA and AEG were detected in the air filters, with no DAB detected from extractions of air filters as a free compound. The amount of particulate BMAA inhaled in August was 0.14 ng/h at both sampling sites (4 and 7). An amount of AEG equivalent to 3.73 ng per hour was detected from an air filter at site 4. When filters were extracted from September and October collections, no free BMAA isomers were recorded via triple quadrupole mass spectrometry. In contrast, impinger samples were more likely to be positive for cyanotoxins than filter samples. At site 4, an amount equivalent to breathing 0.13 ng/h was observed. BMAA as a free amino acid was detected in the impinger samples at both sites in August and at site 7 in October (e.g., Figure 2), with a high concentration of 9.68 ng/h reported at site 7 in August. As a free compound in air, the BMAA isomer AEG was detected at all sites and months, with concentrations ranging between 0.43 and 2.38 ng/h breathed. Although not detected in August, the BMAA isomer DAB was found at all sites in September and October as a free compound, with a range of 0.30 to 0.92 ng inhaled per hour.

At the same time as the air samples were taken, surface samples were collected at air sampling sites and additional locations around the Great Salt Lake to gain a better understanding of the concentrations of cyanotoxins at these locations. Air and surface samples were taken on land as it was considered that with the shore becoming exposed through receding waters in the Great Salt Lake, the prevailing wind would more likely pick up material from solid ground rather than water. The number of sites with active cyanobacteria according to fluorescence microscopy decreased with 5/6, 4/7 and 3/7 sites showing the presence of these organisms in August, September, and October, respectively (Table 2). In ground samples, as a free amino acid, BMAA was only detected in one sample in each of August and September at trace amounts. The frequency of detection and concentration of the BMAA isomers AEG and DAB as a free compound was greater than for BMAA, with DAB the most frequent BMAA isomer detected. Of the three fractions assessed, the free and hydrolyzed free fraction of BMAA in August were found at trace amounts at up to three of the sampled sites, consistent with the presence of cyanobacteria. Similarly, analysis of AEG showed its presence in the three fractions. In the hydrolyzed free fraction, DAB was found in all locations and months with a range between 0.026 to 10.18 ng/mg. In the hydrolyzed bound fraction, BMAA was not detected, with AEG and DAB more often found in the order DAB > AEG > BMAA.

## 3. Discussion

Through global climate change and anthropogenic factors, the waters of the Great Salt Lake are receding, exposing lakebed that once harbored living cyanobacteria. Further effects of climate change are likely to increase the chances of severe storms and wind events which have the potential to make dusts, crusts and dried mats of microorganisms airborne, including cyanobacteria that were present in the Great Salt Lake. Due to the location of the Great Salt Lake and its proximity and orientation to Salt Lake City, Ogden, and other cities of the Wasatch Front, the prevailing wind is likely to transport potential cyanotoxin-containing material over a metropolitan area with over 2.5 million inhabitants. Consequently, an understanding of the types and concentrations of cyanotoxins present in air is needed to begin risk assessment concerning adverse effects to human health.

Cyanobacteria are capable of producing a vast array of bioactive and toxic molecules, including neurotoxins, hepatotoxins and cytotoxins. In acute doses, death or illness can be rapid after exposure to cyanotoxins, with many cases recorded such as the hospitalization of Army recruits in the UK and the deaths of 52 patients at a haemodialysis clinic in Caruaru, Brazil, from exposure to microcystins [50,51,52,53]. Although the acute effects of such compounds are well known, increasing concerns are being raised about long-term chronic exposure to low doses of cyanotoxins. Exposure to microcystins has been implicated in the development of cancer, particularly liver cancer and exposure to BMAA is considered a risk factor for ALS [54]. According to the Bradford-Hill criteria, chronic BMAA exposure is considered to be causative with respect to ALS [55,56].

From the analysis of air samples, BMAA was periodically detected in air as a free compound, showing that airborne exposure is a possibility. The analyses indicated that a person could breathe up 232 ng BMAA per day and was found in air samples at 2 sites in August and 1 site in October. Although insufficient to result in acute toxicity, there is the potential for these toxins to contribute to chronic toxicity. Furthermore, the possibility arises that synergistic toxicity may occur through exposure to multiple toxins [57]. 

BMAA is one of three naturally occurring isomers known to be produced by cyanobacteria. Studies have indicated that both AEG and DAB may be more neurotoxic than BMAA [58]. Consequently, people breathing air near the Great Salt Lake may be inhaling a cocktail of different BMAA isomers. When examining the potential exposome from the Great Salt Lake, as the waters of the lake recede, other components of the lakebed may also become airborne and result in synergistic toxicity. These are largely confined to heavy metals. In GSL sediments nine metals were found to exceed Residential Regional Screening Levels as set by the US EPA [59] and arsenic, lanthanum, lithium and zirconium exceeded EPA Industrial Screening Levels [60]. The lack of correlation between airborne samples and lakebed samples at the same site suggests that other areas west of the Great Salt Lake may contribute to the dust affecting the Wasatch Front. Thus, epidemiological studies on the effect of Great Salt Lake dust on human health are needed [60]. Synergistic effects between metals and cyanotoxins have been well documented. BMAA is known to interact with metals such as copper and zinc and, additionally, microcystins are known to bind metals such as iron [61,62,63]. A further compounding metal problem concerning the Great Salt Lake is that of mercury, which is known to be found in the Great Salt Lake, along with the presence of high concentrations of methylmercury in the deep brine layer creating concerns regarding mercury biomagnification in waterfowl [46]. A better understanding of the components of the aerial exposome derived from the Great Salt Lake is needed to determine the risk(s) to long-term human health.

Two different fractions were analyzed in air samples, the particulate fraction collected on a glass fiber filter along with the presence of free compounds in air, retained in liquid by an impinger. Impinger samples generally contained higher concentrations of BMAA, and its isomers were more frequently detected. Furthermore, some air samples showed the presence of BMAA and isomers in filters and as free compounds. Consequently, with respect to risk exposure and assessment, a better understanding of the different compartments and likely risks that they could pose is needed, including the inhalation of cyanobacteria [64] and free toxin compounds present in air. Furthermore, studies examining atmospheric stress tolerance indicate that cyanobacteria derived from soil do better than those from aquatic habitats [65].

Our analyses of ground samples showed the common presence of BMAA and isomers, frequently and at many sites and their occurrence compared well with the presence of cyanobacteria. When compared with air samples, however, the results from ground samples did not correlate with cyanotoxins in air samples. Other studies examining aerosolized microcystins from lakes have shown little association between recreational exposure to microcystin and noted adverse health effects [66]. Assessment of nasal swabs of individuals showed low microcystin amounts (3.3 ng), but generally more microcystin was found in individuals post-exposure versus pre-exposure at lakes [66]. This preliminary study shows the potential for BMAA and isomers to be present in air samples and further research to fully investigate their presence in air and dried cyanobacterial mats around the Great Salt Lake is warranted. Since air-borne cyanobacteria have been documented as common contaminants on pre-filters of heating, ventilation, and air-conditioning (HVAC) systems with the highest concentrations in autumn [67]), the accumulation of cyanotoxins in residential and commercial buildings is also a concern warranting further research. 

Ultimately, the results of our study suggest that people living on the Wasatch Front may be exposed to multiple cyanotoxins derived from dust resulting from the shrinkage of the Great Salt Lake. Exposure via inhalation could lead to adverse health effects, which are largely thought to be chronic in nature, including an increased risk for ALS and perhaps other progressive neurodegenerative diseases [28,29]. Consequently, a better understanding of the exposure routes, types, concentrations, and synergism with other toxicants derived from the Great Salt Lake is desperately needed. Furthermore, efforts to help restore the water level and conserve the Great Salt Lake have the potential to also protect people from in situ cyanobacteria and their toxins.

## 4. Materials and Methods

### 4.1. Sampling Sites

**Figure 3:**
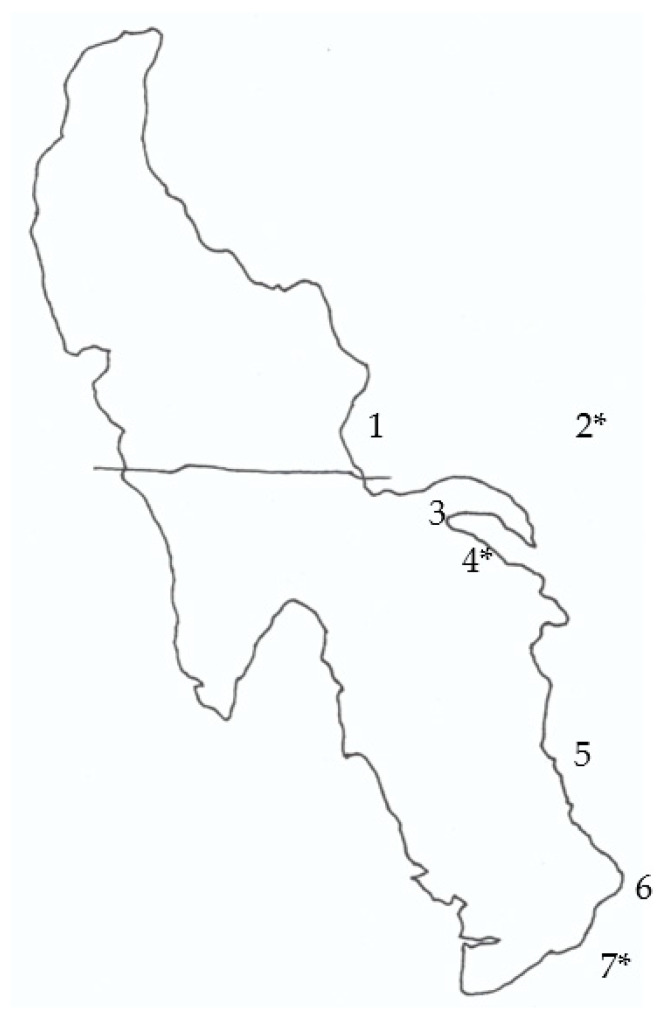
Location of sampling sites around the Great Salt Lake (1–7). *, sites where air samples were taken. The horizontal line across the Great Salt Lake represents the location of the railroad causeway.

Access to sites on publicly accessible land was carried out using a Robinson R44 Raven II helicopter (Blade Helicopters, Ogden, UT) due to the isolation and inaccessibility of many of the sampling sites. Air and/or lakebed samples were taken at publicly accessible sites on the eastern (Promontory Point) down to the southern (Saltaire) areas of the Great Salt Lake (Figure 3). Sampling was performed on 1 day in each month (August, September and October) of 2022 when the lakebed was at its lowest point during the year and without snow cover. After collection, samples were transported to the laboratory on ice.

### 4.2. Air and Ground Sampling

Air samples were not taken at every location due to time constraints. When carried out, a Gilian Aircon 2 air sampling pump was attached to the outlet of a 1 L Erlenmeyer filter flask which acted as an impinger. In the top of the flask a rubber stopper was placed with a hole drilled to allow the placement of a 5 mL plastic Pasteur pipette into the flask. Within the flask, 500 mL of 20 mM HCl were placed. Out of the top of the stopper and attached to the pipette, sufficient plastic tubing was placed to allow attachment to a housing containing a 40 mm GF/C filter (Whatman, Cytiva, Marlborough, NZ, USA). The side arm from the Erlenmeyer flask was attached to the air pump. As sampling sites were remote, the Gilian Aircon 2 pump was powered by a portable power station (Jackery Explorer 500, Amazon.com), supplemented with a portable solar panel (Jackery SolarSaga 100W, Amazon.com). At each location the air pump was run for approximately 2 h at a flow rate of 4 L per minute, roughly equal to 500 L of air passing through the pump and equivalent to the amount of air breathed by an average person in 1 h [44].

### 4.3. Water and Lakebed Sampling

At every location, representative ground samples were taken. Solid samples were collected using a trowel and placed in zip-lock bags with liquid samples placed in 50 mL plastic centrifuge tubes. All samples were then placed in a cooler until processed at the laboratory.

### 4.4. Laboratory Processing of Samples

Samples received at the laboratory were kept cold. Environmental collections of lakebed and water underwent fluorescence and light microscopy to determine the presence of cyanobacteria using an Axioplan 2 microscope (Zeiss, Jena, Germany) before being frozen and freeze-dried. 

The GF/C filters were observed for any superficial material and freeze-dried. The Erlenmeyer impinger samples were removed and underwent solid phase extraction (SPE). An in-series set of SPE cartridges was prepared using a C18 cartridge (Biotage, Uppsala, Sweden) before a SCX SPE cartridge (Phenomenex, Torrance, CA, USA). The system was primed with 20 mL methanol, followed by 20 mL 20 mM HCl. The contents of the impinger liquid were allowed to pass through the two-cartridge system and the eluate was collected. The SCX cartridge was eluted with 10 mL 2% (*v*/*v*) NH_4_OH/methanol and dried in a rotary evaporator. The residues were resuspended with 1 mL 20 mM HCl and placed in an Eppendorf before storage at −20 °C.

### 4.5. Extraction of Cyanotoxins from Great Salt Exposed Lakebed Samples

Concerning BMAA extraction, subsamples of the freeze-dried material were weighed and resuspended with 20% (*w*/*v*) trichloroacetic acid (TCA). Samples were sonicated and stored overnight at 4 °C. The following day the samples were warmed to room temperature and centrifuged. The supernatant was removed and placed in a microcentrifuge tube and an aliquot underwent centrifuge filtration at 10,000× rpm (Spectrafuge 16M, VWR, Radnor, PA, USA) before the filtrate was stored at −20 °C (Free). A further aliquot of the TCA extraction was mixed with an equal volume of 12M HCl and underwent hydrolysis at 110 °C for 16 h. The following day, the digest was allowed to cool, and an aliquot again underwent centrifuge filtration, and the filtrate was stored at −20 °C until analysis (hydrolyzed free). A volume of 6M HCl was added to the remaining pelleted material after TCA extraction, and the suspension underwent hydrolysis at 110 °C for 16 h. The following day, the digest was also allowed to cool, and an aliquot again underwent centrifuge filtration, and the filtrate was stored at −20 °C until analysis (hydrolyzed).

### 4.6. Cyanotoxin Analysis

The frozen BMAA extracts were allowed to thaw and were diluted with purified water (≥18 MΩ) before derivatization with 6-aminoquinolyl-N-hydroxysuccinimidyl carbamate (AQC) alongside standards of BMAA and isomers as previously published [68]. This method is fully validated for the assessment of BMAA and isomers in cyanobacterial matrices and in 20 mM HCl, suitable for the assessment of these toxins in air. Multiple sample dilutions were tested in order to optimize the AQC derivatization reaction. A stable BMAA isotope internal standard (C_4_ H_7_^2^H_3_^15^N_2_ O_2_) was added to all samples and standards for analysis. The assay itself had a limit of detection (LOD) of 10 pg/mL, a lower limit of quantification (LLOQ) of 37 pg/mL and a working range of 0.002 to 153 ng/mL.

## Figures and Tables

**Table 1:** Concentrations of free cyanotoxins in air filter and impinger samples from around the Great Salt Lake.

Sampling Location	BMAA Filter	AEG Filter	DAB Filter	MC Impinger	BMAA Impinger	AEG Impinger	DAB Impinger
	ng *	ng *	ng *	ng *	ng *	ng *	ng *
**Aug. 2022**							
Site 4	0.14	3.73	ND	0.13	0.33	0.56	ND
Site 7	0.14	ND	ND	NQ	9.68	0.43	ND
**Sept. 2022**							
Site 2	ND	ND	ND	ND	ND	2.38	0.92
Site 4	ND	ND	ND	ND	ND	1.82	0.58
Site 7	ND	ND	ND	ND	ND	0.84	0.52
**Oct. 2022**							
Site 2	ND	ND	ND	ND	ND	1.08	0.40
Site 4	ND	ND	ND	ND	ND	0.98	0.30
Site 7	ND	ND	ND	ND	0.35	1.21	0.40

For site locations, see Figure 2; * Amount of toxin likely inhaled per hour; ND, not detectable, NQ, not quantified; ng, nanograms.

**Table 2:** Mean cyanotoxin concentrations in ground samples from around the Great Salt Lake.

		Free (ng/mg)			Hydrolysed Free (ng/mg)			Hydrolysed (ng/mg)		
Site/Time	Cyano	BMAA	AEG	DAB	BMAA	AEG	DAB	BMAA	AEG	DAB
**Aug. 2022**										
1	+	ND	ND	0.212	0.004	0.007	2.214	ND	0.973	1.248
3	+	ND	0.217	0.217	0.002	0.004	2.333	ND	0.251	1.120
4	+	0.005	0.134	0.580	ND	ND	3.693	ND	1.913	4.925
5	+	NQ	ND	0.074	0.002	0.004	1.724	ND	0.119	1.498
6	−	ND	ND	0.001	ND	ND	0.026	ND	ND	ND
7	+	ND	ND	0.017	ND	ND	1.347	ND	ND	0.003
**Sept. 2022**										
1	+	ND	ND	0.170	ND	ND	2.394	ND	ND	6.272
2	−	ND	0.843	0.032	ND	ND	0.187	ND	ND	2.696
3	+	ND	0.032	1.489	ND	ND	10.181	ND	0.061	4.218
4	+	ND	0.900	0.176	ND	ND	1.772	ND	0.882	1.135
5	+	ND	ND	0.031	ND	ND	0.924	ND	ND	0.001
6	−	ND	NQ	0.010	ND	ND	0.138	ND	ND	0.201
7	−	0.009	ND	0.003	ND	ND	0.219	ND	ND	0.002
**Oct. 2022**										
1	+	ND	ND	0.062	ND	ND	1.747	ND	ND	0.047
2	−	ND	ND	ND	ND	ND	0.148	ND	NQ	0.078
3	+	ND	ND	0.065	ND	ND	3.544	ND	NQ	1.382
4	+	ND	ND	0.646	ND	ND	0.077	ND	ND	2.525
5	−	ND	ND	0.002	ND	ND	0.513	ND	ND	ND
6	−	ND	ND	ND	ND	ND	0.098	ND	NQ	0.052
7	−	ND	ND	0.009	ND	ND	1.507	ND	ND	ND

ND, not detected; NQ, not quantified.

## Data Availability

Please contact the authors.
